# Using machine learning to forecast domestic homicide via police data and super learning

**DOI:** 10.1038/s41598-023-50274-2

**Published:** 2023-12-21

**Authors:** Jacob Verrey, Barak Ariel, Vincent Harinam, Luke Dillon

**Affiliations:** 1 Institute of Criminology, University of Cambridge, Sidgwick Ave, Cambridge, CB3 9DA UK; 2 Institute of Criminology, The Hebrew University of Jerusalem Mt. Scopus, 9190501 Jerusalem, Israel

**Keywords:** Engineering, Human behaviour, Software, Statistics

## Abstract

We explore the feasibility of using machine learning on a police dataset to forecast domestic homicides. Existing forecasting instruments based on ordinary statistical instruments focus on non-fatal revictimization, produce outputs with limited predictive validity, or both. We implement a “super learner,” a machine learning paradigm that incorporates roughly a dozen machine learning models to increase the recall and AUC of forecasting using any one model. We purposely incorporate police records only, rather than multiple data sources, to illustrate the practice utility of the super learner, as additional datasets are often unavailable due to confidentiality considerations. Using London Metropolitan Police Service data, our model outperforms all extant domestic homicide forecasting tools: the super learner detects 77.64% of homicides, with a precision score of 18.61% and a 71.04% Area Under the Curve (AUC), which, collectively and severely, are assessed as “excellent.” Implications for theory, research, and practice are discussed.

## Introduction

Domestic abuse is a substantial problem in the United Kingdom and across the globe. In the UK alone, it affected 2.4 million adults for the year ending in March 2022^1^, cost the British government £68 billion annually^2^, and inflicted psychological damage on children, families, and communities that is difficult to quantify^3–5^. Perhaps the most insidious form of domestic abuse is domestic homicide, or a form of domestic abuse that results in death. Indeed, a Home Office report estimates that a single case of domestic homicide costs the UK £2.2 million on average^2^, with domestic homicides accounting for nearly a fifth of all police-reported homicides^1,6–8^.

It is no surprise that police in the United Kingdom consider the prevention of domestic homicide a top priority. The London Metropolitan Police Service (MPS) is the UK’s largest police force that is estimated to respond to 25 cases of domestic homicide per year (see supplemental materials for estimate computation)^9,10^. With each case costing £2.2 million^2^, these domestic homicide cases translate to an annual loss of £55 million, and they also inflict profound psychological damage on those affected by the homicide^3–5^.

Unsurprisingly, the MPS has a demonstrated interest in preventing domestic homicide, and they have attempted to do so via forecasting. Forecasting is often the first step in crime prevention^11^; it involves predicting who will commit a crime so that police can attempt to stop them, and it typically demands access to a large amount of data. To implement forecasting, the MPS has amassed a rich internal dataset of domestic abuse cases as well as a risk assessment system. Unfortunately, the MPS’s current forecasting procedure has been deemed weak (P. Neyroud, pers. comm., May 25, 2023), and calls are made to identify alternative, more advanced prediction instruments based on artificial intelligence^12^.

Machine learning can be used to create these prediction instruments. Specifically, machine learning is a branch of artificial intelligence in which the computer learns patterns in data^13^. It has shown great promise in multiple criminal justice studies^14–18^, including domestic abuse^19^, and it has a demonstrated history of producing valid forecasting instruments^20^. Can the MPS—or police departments globally—use machine learning to improve the forecasting accuracy of domestic homicide? This question is presently unanswered.

This paper aims to investigate the utility of machine learning in domestic homicide forecasting using existing police records. Specifically, the article applies innovations from machine learning—the implementation of a “super learner”—to the MPS’s domestic abuse dataset to illustrate its utility in forecasting domestic homicides. To further illustrate its utility, the performance of the super learner will be compared to the performance of the MPS’s current risk-rating system, as well as the performance of two other state-of-the-art domestic homicide forecasting tools: the Lethality Assessment Programme and the Danger Assessment (see supplemental materials for review)^21–23^. This super learner’s implementation may therefore help police across the globe detect domestic homicides before they turn fatal, using their own data, thereby preventing them and mitigating this costly and insidious crime. It may also replicate earlier findings on super learning, a type of ensemble learning, illustrating how it can be applied to this new type of dataset.

## Results

### Super learner outperforms all individual machine learning models

The super learner ensemble should be able to either outperform or perform just as well as any individual machine learning model that went into its construction^24,25^. To test this assertion, the performance of the entire super learner was compared to the performance of all the individual models used to construct it, and the results appear in Table 1. This assertion was upheld: the super learner was the single best-performing model on the MPS dataset, producing an AUC score of 0.7104, whereas the next best-performing model produced an AUC score of 0.6681.

**Table 1:** The performance of super learner compared to performance of individual models used to assemble it.

Model	Recall	Precision	Specificity	AUC
**Super learner**	**0.7764**	**0.1861**	**0.6443**	**0.7104**
Extra trees	0.6500	0.2006	0.7212	0.6856
AdaBoost	0.6500	0.1997	0.7203	0.6851
Decision tree	0.6543	0.182	0.6818	0.6681
Support vector machine	0.6908	0.1799	0.6451	0.6679
Random forest	0.6154	0.1883	0.718	0.6667
Logistic regression	0.5556	0.197	0.6424	0.599
Linear discriminatory analysis	0.5259	0.1621	0.6667	0.5963
Gradient boosting	0.3976	0.1744	0.7256	0.5616
AdaBoost*	0.1505	0.4174	0.9124	0.5315
K Nearest neighbor	0.1089	0.1225	0.9368	0.5228
Gaussian Naïve Bayes*	0.9705	0.0975	0.0592	0.5148

Models are sorted by descending AUC score. All models were constructed using the optimized hyperparameters unless indicated by an asterisk, in which case they are used the default. The super learner is bold. Full details of how these models were obtained appears in the supplemental materials.

### Super learner outperforms MPS’s risk assessment system

**Figure 1:**
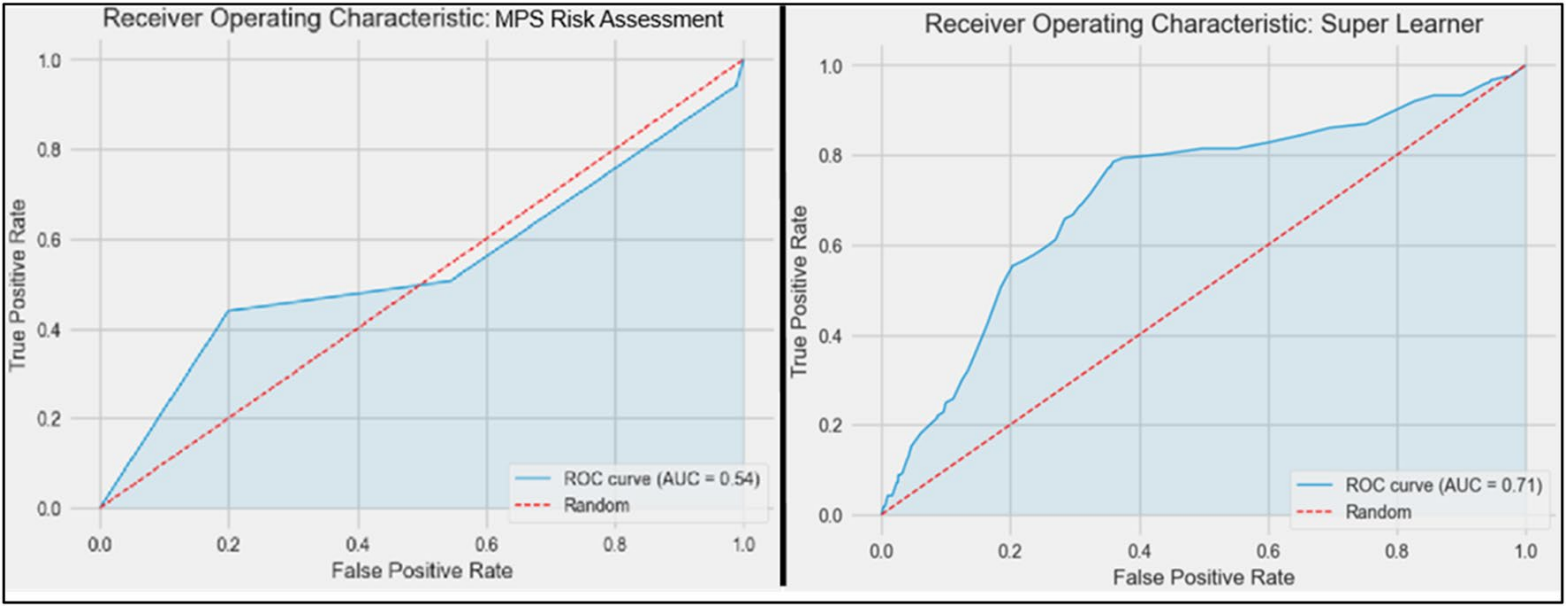
ROC Curves. The London Metropolitan Police Service’s (MPS) standard risk assessment appears on the left, whereas the super learner appears on the right.

The super learner dramatically improves the MPS’s unvalidated risk assessment: its recall score is nearly double that of the MPS, its precision score experienced no meaningful decrease, and its AUC score has improved substantially. Using AbiNader et al.’s scoring criteria^26^, the super learner has produced the model with a score of “excellent” whereas the MPS’s model appears little better than chance. The full results appear in Table 2, with the ROC curves appearing in Fig. 1.

**Table 2:** Super learner versus other domestic homicide forecasting tools.

Tool	Use case	Victim gender	Recall	Precision	Specificity	AUC
Super learner	Police	Male or female	.78	.19	.64	.71
MPS Risk assessment	Police	Male or female	.44	.19	.80	.54
Lethality assessment Programme^21,22^	Police	Female only	.93	.13	.21	.37
Danger assessment^23^	Medics	Female only	.83	.25	.56	.79

### Super learner outperforms the lethality assessment programme

The super learner has a recall score fifteen percentage points lower than the Lethality Assessment Programme, meaning it can detect fewer homicides. However, the super learner produces fewer false positives, as evident by its superior precision and specificity scores, suggesting that its predictions are more reliable. Given this ambiguity, the AUC scores were used to compare the two models. The super learner, which has an AUC score of 0.7104, outperforms the Lethality Assessment Programme, which has a score of 0.3747.

### Super learner underperforms the danger assessment, but underperformance may be immaterial

The Danger Assessment reports a recall and precision score a few percentage points greater than the super learner, meaning it can detect more homicides while producing fewer false positives. Despite its superior performance, however, the Danger Assessment is limited in that it can only be used on female victims, and it may also be incompatible with policing data—observations that are discussed in the discussion.

## Discussion

Domestic homicide is an insidious and costly crime for London and the United Kingdom^1,2,10^. If these incidents can be forecasted, then they are potentially preventable^11^. In this paper, we created a domestic homicide forecasting tool via machine learning, built strictly from police records. We limited ourselves to police records because these types of data are used in routine police operations aimed at detection and prevention^27^. Specifically, this study applied van der Laan et al.’s super learner paradigm to the MPS’s dataset^24^. In the process, our super learner made domestic homicide forecasting predictions that (i) outperform the MPS current risk assessment procedure and (ii) outperform any other material domestic homicide forecasting tool. Implications are discussed below.

### Super learner outperforms all material domestic homicide forecasting tools with police data

The super learner outperforms all tools except the Danger Assessment. Yet, this underperformance may be immaterial because the Danger Assessment suffers from two major limitations: it cannot be easily applied to male victims, and it is unsuitable for police data. Regarding the former, males represent roughly a quarter of all domestic abuse victims in both the United Kingdom and the MPS’s dataset^1^. Unfortunately, the Danger Assessment cannot screen cases in which there was a male victim, meaning it is unable to screen the nearly 700,000 cases of domestic abuse that occurred in the year ending in March 2022 involving a male victim^1^. Second, the Danger Assessment was designed to ask intimate questions in a medical setting^28^. It was unclear if those with access to police data could use this tool, however, later researchers translated this tool into something officers could use after dramatically redesigning it^21,22^. This redesign heavily suggests that there were issues implementing the initial tool in a police setting. The super learner was built from policing data, so if those in the policing space cannot use the Danger Assessment, then its performance may not be material.

### Replication and translation of super learning into other areas of law enforcement

Outside of creating a domestic homicide forecasting model, this study also served replicates van der Laan et al.’s original assertion that the super learner should either outperform or perform just as well as the individual models that went into its creation^24^. Moreover, this study can serve as a proof-of-concept for how the super learning paradigm can be applied to other policing datasets. We envisage, for example, the utility of this instrument for identifying missing persons at risk of harm, gang related violence, and spatial hotspots of crime, among other use cases^29^.

### Potential to prevent domestic homicide

Finally, domestic homicides are a costly and insidious crime: they cost the UK £2.2 million per case and inflict profound psychological damage on communities of people affected by the homicide^2–5^. If the super learner is used to help officers prevent a domestic homicide—even if it is just a single case—then it represents a serious cost-saving and lifesaving opportunity for the United Kingdom. Moreover, many domestic abuse victims experience multiple instances of domestic abuse^2^. The more an offense happens, the more likely police can intervene. Therefore, the multiple recurring instances of domestic abuse give police multiple opportunities to intervene. Police can use the super learner during these interventions to predict whether the abuse will turn fatal, and if so, then police can stop the cycle of abuse and potentially save a life.

### Policy implications

First, it is important to wrestle with the ethical challenges of using machine learning on a police dataset. In other words, features from policing data are prone to both errors and biases against certain groups^30^. Therefore, we recommend adding additional features to the super learner from diverse datasets that are less susceptible to unfairness. Moreover, records that can be added retrospectively—such as medical records, employment history, and postal codes—will almost certainly improve the super learner’s prediction validity, in addition to mitigating possible dataset-related biases. Indeed, one might suggest that the more relevant data one adds, the better the super learner’s predictions will become—a suggestion that can be executed infinitely insofar as much as ethics will allow.

Third, once the super learner has reached an asymptomatic performance, one can use precision, recall, and AUC scores to determine how to best use the model. If the precision remains relatively low—meaning false positives remain high—then one ought to continue to use the model as suggestive. However, if precision increases—if the model gets to the point where most names it highlights *will* commit a domestic homicide—then perhaps its output should be given more weight over purely clinical decision-making models.

Additionally, when deploying a machine learning system, it is crucial for those operating the system to be trained in machine learning. This is because operators—like many members of the public^31^—may have a cognitive bias in which they rate a computer’s judgment higher than their own. This is extremely problematic because machine learning models—like the super learner—may have a significant false positive rate, meaning that, if a model flags a case as elevated risk, people might be prone to treating the case more severely despite their judgment telling them otherwise. Training can be used to mitigate cognitive biases^32^, and thus, education on machine learning evaluation can be used to inoculate operators from such a bias.

## Limitations

### High false positive rate

The predictions of the super learner should be interpreted as suggestive rather than deterministically. In other words, its high rate of false positives suggests it can highlight cases with an *elevated* risk of domestic homicide, yet it cannot be used to suggest that a case *will* result in a domestic homicide. Specifically, for every five cases a super learner highlights, only one of these cases will result in a domestic homicide; the remaining four are false positives. Once again, this output is still an improvement over the existing instruments for domestic homicide forecasting, but these predictions must be interpreted appropriately.

The high rate of false positives is undesirable, so a few measures can be taken. First, individuals can add more data, and this would likely improve the performance of the super learner^33^. Second, the models in the super learner can be re-optimized so that they maximize precision at the expense of recall. This would produce a far higher precision score—perhaps as high as 0.5—yet this would come at the expense of dramatically fewer homicide predictions.

### Unideal dataset

Ideally, the study would have used a dataset with a more robust label. In other words, the homicide label within the MPS dataset contains cases that *were* homicides; these are not cases that *will become* homicides. This suggests that the tool cannot be used for out-of-the-box deployment; it should be re-trained and replicated on a dataset that contains the latter. Moreover, the super learner was only configured to screen the most serious cases of domestic abuse, i.e., cases that may most likely become a homicide. Because of this, the super learner cannot screen all domestic abuse cases, though this should be read as a trade-off rather than a limitation. Specifically, police in London respond to an average of 7595 domestic abuse offenses per month, and they may also face a new age of austerity where they are asked to do more work with fewer resources^34,35^. They may not have the resources to run thousands of domestic abuse cases through a super learner every month; they could use their resources much more efficiently by focusing only on the most serious cases, or those most likely to escalate. The trade-off is that they cannot forecast homicide from less-serious cases, yet this trade-off can be reversed if the super learner is re-trained on more cases.

Notwithstanding these limitations, this study still showed that the super learner can be applied to police data, resulting in a performance increase when compared to other machine learning models and current police practice. Moreover, insofar as much as the MPS dataset represents the typical policing dataset, the same super learning finding can be generalized to other datasets for a similar performance increase. Indeed, many criminal justice agencies use machine learning, albeit they are using far less powerful, sophisticated algorithms^14,16,36^. This paper may suggest that, if they were to implement super learning, they would receive a material performance increase—a finding that likely holds regardless of the impurities of the MPS dataset.

### Deployment

While the study successfully created a forecasting model, it did not address how police should use it in practice. In other words, it is unclear how officers should respond to a forecasted homicide. They can, for example, deploy a proportional intervention such as focused deterrence, or they can use this information to implement civil rights concerns^2,37–39^.

Addressing these societal concerns is well beyond the scope of the present investigation; however, it is essential to note that forecasting tools such as this have been used in the criminal justice space for nearly a century^40,41^. Indeed, as far back as 1928, practitioners have been using tools to forecast events like recidivism, yet not all of these tools were based on data; many were based on intuition, and as a result, they were susceptible to poor performance. Statistical tools were later developed^41^, yet these tools were limited to the confines of the generalized linear models they inherited from, and thus, their predictive accuracy suffered^42^. When viewed in this light, machine learning tools are simply the next iteration of a century-long tradition of using tools to forecast criminal-justice-related events. These tools have practices surrounding their deployment, so for deployment guidance, police can build off the century-long tradition of deploying such tools in the criminal justice space.

### Potential for bias

This study was focused on producing the best model possible, as determined by the AUC score; it was not concerned with producing the fairest model possible. In other words, policing data—especially that which contains professional judgment—has been known to contain racial biases^30^, and thus, the model could have been trained on racially biased data. If this was the case, then the model would accentuate these racial biases, yet this concern could be mitigated via proper machine learning techniques^43,44^.

Unfortunately, these racial-prejudice-mitigating procedures were not undertaken in part because they sometimes result in poorer model performance^43,45^. However, this limitation highlights a deeper issue with the domestic homicide forecasting literature: these tools are judged for their performance, not for their fairness^21–23,28,46^. In other words, the key performance indicators used to evaluate these models score the *robustness* of the model’s predictions; they don’t score the model’s *fairness*, and if the fairness isn’t scored, then it cannot be evaluated. Moreover, the super learner is relatively opaque, meaning it is difficult to assess these fairness measures. Thus, while fairness is an important concern, it remains largely unaddressed in the forecasting literature, and for this reason, it is beyond the scope of the present study.

## Methods

### Ethics

All methods were performed in accordance with relevant guidelines, regulations, and protocols. The Ethics Committee of the Institute of Criminology, University of Cambridge approved of all aspects of this experiment, including all protocols, guidelines, and regulations followed, as well as provided ethical oversight. Moreover, this study analyzed legacy crime data collected in a standard policing process. Due to the retrospective nature of the study, the need for informed consent was waived by the Ethics Committee of the Institute of Criminology, University of Cambridge. MPS fully anonymized these data before handing them to the authors.

### Dataset overview and description

The MPS’s internal crime database was used to obtain the present dataset. First, the MPS database was queried for all domestic abuse cases between January 1, 2009, and December 31, 2019. Second, these cases were further screened so that only the most serious type of domestic abuse case appeared in the dataset: any case that involved murder, attempted murder, conspiracy to commit murder, poisoning, or intentional grievous bodily harm was included. Finally, any of these cases that involved a homicide were flagged, resulting in a grand total of 2500 cases: 2,263 non-homicide and 237 homicides.

The dataset contains thirteen features that describe details behind the offense, the offender, and the victim, with the ultimate label being whether the case was a homicide. A full description of each feature, as well as descriptive statistics, appears in Suppl. Tables 1–2. Overall, the gender and ethnic composition of victims in this London dataset is representative of their equivalent in the UK^1,47^.

## Preprocessing and feature engineering

Five key data modifications were made to convert these features into a format conducive to machine learning: ordinal encoding, one-hot encoding, standardization, the removal of irrelevant features, and the removal of redundant features^48^. Details appear in the supplemental materials.

## Model construction and evaluation

### Overview

**Figure 2:**
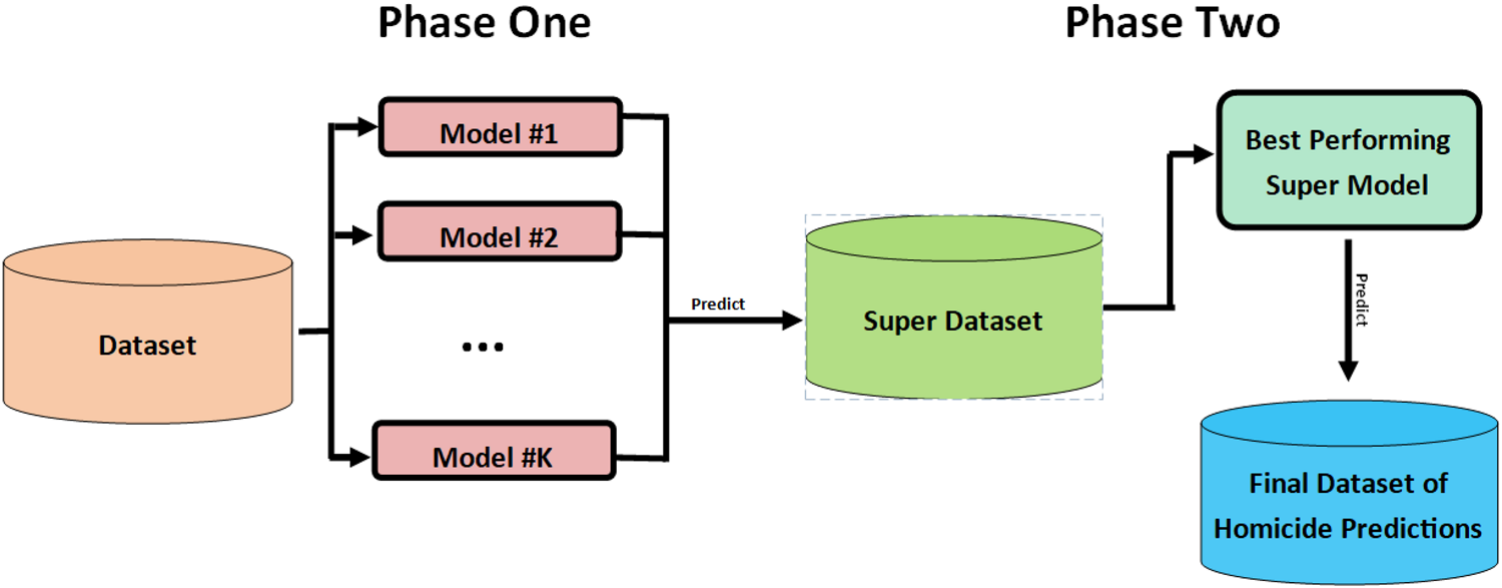
Illustration of the Super Learner. *Phase One* depicts a dataset being ran through various machine learning models, which each independently predict whether an individual will commit a domestic homicide. The results of these multiple predictions are stored in the Super Dataset. *Phase Two* depicts a separate machine learning model—the super model—generating one final homicide prediction from the super dataset—a dataset that contains the prediction of other machine learning models.

The super learner paradigm, also known as meta learning, is a type of ensemble learning that stacks or combines the prediction of multiple models^24,25^ Similar to the wisdom of the crowds^49^, the final prediction of the super learner is often greater than the sum of its parts: the final super learner tends to outperform the individual machine learning models that went into its prediction. A diagram of the super learner appears in Fig. 2.

Following Fig. 2, the super learner was implemented in two phases. In phase one, a series of initial models were trained and validated on the MPS dataset via ten-fold cross-validation. These models independently predicted whether each out-of-sample case was a homicide; their predictions were stored in the “super dataset,” a dataset of predictions. In the second phase, a model was trained on the super dataset. Its purpose was to intelligently combine the predictions in a way that produced a homicide prediction that was greater than the sum of its parts. However, it is difficult to know which model will perform best on the super dataset a priori^24,25^, so several different models were trained and validated. The precision, recall, specificity, and AUC scores were recorded for all models, with the AUC score being the study’s primary evaluation metric. Details of these evaluation metrics appear in supplemental materials.

### Phase one: initial models

Ten initial machine learning classifiers were used for the present homicide forecasting task: a Logistic Regression^50^, the CART Decision Tree^51^, Random Forest^52^, Extra Trees^53^, Gradient Boosting Classifier^54^, Adaptive Boosting (“Ada boost”) Classifier^55^, K Nearest Neighbors^56^, Linear Discriminant Analysis^57^, Gaussian Naïve Bayes^56^, and a Support Vector Machine^58^.

First, each classifier was trained to produce a preliminary model; they were evaluated on the entire MPS dataset via ten-fold cross-validation, and their precision, recall, and AUC scores were extracted. Second, their hyperparameters were tuned to maximize their AUC score, this study’s primary evaluation metric. Third, precision, recall, specificity, and AUC scores were obtained for the newer, optimized models. Each optimized model outperformed the preliminary model, as determined by the AUC score. Details of this full procedure appear in the supplemental materials: the full results of each model appear in Suppl. Table S4, whereas the full results of the hyperparameter tuning appear in Suppl. Table S6.

### Phase two: super learner

To construct the super learner, the models from phase one made out-of-sample predictions on the entire dataset via ten-fold cross-validation; their predictions were saved to the super dataset. Second, each of the ten classifiers from the above subsection was re-trained on the super dataset; they each produced a model whose AUC, precision, recall, and specificity scores were extracted via ten-fold cross-validation. Third, the hyperparameters of these classifiers were optimized on the super dataset to produce the highest possible AUC score. Fourth, the optimized classifiers were re-trained on the super dataset to build an optimized model. Finally, the best-performing model—the optimized decision tree—was selected as the ultimate super learner. The entire super learner is now complete, and the precision, recall, specificity, and AUC scores were obtained for the entire apparatus. Full details of this procedure appear in the supplemental materials: the results of each model appear in Suppl. Table S5, whereas the full results of the hyperparameter tuning appear in Suppl. Table S6.

## Contextualization

To contextualize the performance of the super learner, it was compared to the MPS’s current risk assessment procedure, as well as two state-of-the-art domestic homicide forecasting techniques: the Danger Assessment and the Lethality Assessment Programme. Specifically, the recall, precision, specificity, and AUC scores were extracted for all three instruments and compared to the present super learner. Details concerning the identification and extraction of these scores appear in supplemental materials.

### Supplementary Information

Supplementary Information.

## Data Availability

Upon reasonable request, the anonymized dataset can be made available subject to a non-exclusive, revocable, non-transferable, and limited right to use the data for the exclusive purpose of undertaking academic research, subject to approval by the London Metropolitan Police Service. Please address all requests to the corresponding author.
