# Supplementary material for: Using machine learning to forecast domestic homicide via police data and super learning
Source: Sci Rep. 2023 Dec 21;13:22932. doi: 10.1038/s41598-023-50274-2 (PMC10739734; doi:10.1038/s41598-023-50274-2)
Supplement: Supplementary file 1 — Supplementary Information. [file 41598_2023_50274_MOESM1_ESM.pdf]

SUPPLEMENTAL MATERIALS

Using Machine Learning to Forecast Domestic Homicide via Police Data and Super Learning

Jacob Verrey[1*], Barak Ariel[2], Vincent Harinam[3], Luke Dillon[4]

[1] Cantab. Institute of Criminology, University of Cambridge, Sidgwick Ave, Cambridge CB3 9DA, +44 1223 335360; jjv31@cam.ac.uk

[2] Professor of Experimental Criminology, Institute of Criminology, University of Cambridge, Sidgwick Ave, Cambridge CB3 9DA, +44 1223 335360, ba285@cam.ac.uk; and Associate Professor of Criminology, Institute of Criminology, The Hebrew University of Jerusalem Mt. Scopus, Jerusalem, Israel, 9190501, barak.ariel@mail.huji.ac.il

[3] PhD, Institute of Criminology, University of Cambridge, Sidgwick Ave, Cambridge CB3 9DA, +44 1223 335360, vh315@cam.ac.uk

[4] Cantab. Institute of Criminology, University of Cambridge, Sidgwick Ave, Cambridge CB3 9DA, +44 1223 335360; Metropolitan Police Service, ltod2@cam.ac.uk

---

* Corresponding author. *Email:* jjv31@cam.ac.uk (J. Verrey).

Supplemental Introductory Materials

Estimating Domestic Homicides in London

The London Metropolitan Policing Services ("MPS") does not report cases of domestic homicides via its publicly available datasets.[1] However, MPS did report that they experienced an average of 132.5 homicides per year during the four-year period between 2018 and 2022. Domestic homicides encompass 19.03% of all homicide cases across the United Kingdom.[2–5] When applying these macro-level statistics to the MPS annual homicide cases, we estimates that 25 of these 132.5 homicides are domestic.

Existing Domestic Homicide Forecasting Tools

Domestic homicide forecasts are often conducted via screening tools.[6]. These screening tools do not use machine learning; they are typically questionnaires administered to a victim of domestic abuse, and they assign the victim a risk category (e.g., high, medium, or low). In a state-of-the-art literature review, Graham and colleagues identified 18 different screening tools that were administered in eight countries applied by various professional groups (law enforcement, social workers, academics, researchers, etc.).[7] Most screening tools did not forecast domestic homicide; they predicted whether an individual would suffer from a future, non-lethal domestic abuse incident.[8] However, these tools cannot be used in this study because factors that predict domestic abuse may differ from those that predict domestic homicide.[9] All abuse is damaging, but there are variations between different types of assaults, threats, and violence that perpetrators inflict on their victims. Severity scores can be used as indexes that enable a more nuanced classification of offenders. This can then be used to create typologies of domestic abuse pathologies, such as coercion, pathological behavior, sadism, or homicidal behavior.[10,11] Critically, research shows that there are phenomenological differences

between domestic homicide and non-lethal domestic abuse in terms of the "emotions that trigger it, the circumstances that led up to it, and the state of mind that characterizes it."[12] Therefore, predicting non-lethal repeat victimization is not necessarily similar to predicting lethal domestic abuse.

Still, we identified two instruments that attempted to forecast near-fatal domestic abuse, or domestic abuse incidents in which the victim was nearly killed—the Danger Assessment and Lethality Assessment Programme—which are described below.

The Danger Assessment. The first tool that forecasts near-fatal domestic abuse is the Danger Assessment. Developed by Campbell,[13] this questionnaire was published in *Advances in Nursing Science* and is intended to be administered in a medical setting. It consists of 20 weighted questions and assigns the respondent a risk category. The survey is calibrated for female victims of domestic abuse, so it may not be fitting for male victims (e.g., the instrument queries about strangulation, abuse during pregnancy, etc.).[14]

It is difficult to measure the performance of this tool because the original validation study did not report false discovery or precision rates.[14] Nearly all of the other studies in Graham et al.'s review report on these crucial model performance indicators, and without them, it is difficult to determine how well the Danger Assessment is performing.[7] Fortunately, a related study found that a modified Danger Assessment can predict 83% of near-fatal domestic abuse with a false discovery rate of 75%.[15] In other words, only one of the four women the model identified as being at exceptionally high risk would actually die at the hands of her abuser. Other studies have received similar results when they used this tool to predict less extreme manifestations of domestic abuse,[16–18] suggesting these findings have been somewhat replicated.

Lethality Assessment Programme. The second forecasting tool is a modified version of the Danger Assessment created for law enforcement by Messing and colleagues.[19,20] Specifically, the US Department of Justice funded the original validation studies, and they were conducted through

various law enforcement collaborations with officers in Oklahoma, United States. This tool reduces Campbell et al.'s original 20 questions to 11, and it desensitizes them so that they can be asked by an officer responding to a domestic abuse call-for-service.[14] Like the tool it inherits from, the Lethality Assessment can only screen women rather than men. The original validation studies suggest that it can detect 93% of all near-fatal domestic abuse, with an 87% false discovery rate. However, there are no replications as of 2021.[7]

Supplemental Methods and Results

Dataset Description

Describe statistics that describe the categorical (Table S1) and continuous (Table S2) features appear below. Information concerning each feature can be inferred from its name, except for the initial risk assessment. The initial risk assessment was conducted according to the guidelines outlined in the Domestic Abuse, Stalking, Harassment and Honour-Based Violence Assessment (DASH) programme. This was the most widely used domestic abuse risk assessment system in the United Kingdom.[21]Specifically, the programme required officers to ask a series of questions to the victim of a suspected domestic abuse case. Then officers would rank a case as high, medium, or standard risk.[22]

DASH is not a forecasting instrument; it is simply meant to assess whether there is a risk of serious harm.[22] Moreover, unlike previous forecasting instruments,[7] it is not a quantitative risk-rating system: it is a heuristic instrument risk rating. The questions are meant to inform an officer's intuition, but there are no numeric guidelines to guide officers in assigning a risk rating. For this reason, these risk ratings may have considerable variation between officers and over time.

Table S1. Descriptive statistics of categorical features and label in MPS dataset. The label is marked with an asterisk (*)

Feature	Count	Percentage
**Suspected Offender Sex**		
Male	1909	76.36%
Female	591	23.64%
**Ethnic Appearance (Offender)**		
White – Northern European	1047	42.28%
White – Southern European	199	7.68%
White European	37	1.48%
Black	820	32.80%
Afro-Caribbean	4	0.16%
Middle Eastern	37	1.48%
Arab	1	0.04%
Asian	306	12.24%
Chinese, Japanese, or SE Asian	23	0.92%
Unknown	16	0.64%
**Initial Risk Assessment**		
High	555	22.20%
Medium	798	32.92%
Small	1106	44.24%
Unknown	41	1.64%
**Suspect Relationship with Victim Summary 1**		
Boy/Girlfriend	1878	75.12%
Partner/Spouse	622	24.88%
**Suspect Relationship with Victim Summary 2**		
Current	1635	65.40%
Ex	865	34.60%
**Suspect Relationship with Victim (Detailed)**		
Boyfriend	774	30.96%
Ex Boyfriend	631	25.24%
Husband	356	14.24%
Girlfriend	321	12.84%
Ex Girlfriend	144	5.76%
Wife	129	5.16%
Ex Husband	68	2.72%
Common Law Husband	33	1.32%
Common Law Wife	10	0.40%
Ex Wife	9	0.36%
Same Sex – Civil Partner	7	0.28%
Ex Common Law Husband	6	0.24%
Same Sex - Intimate Partner	5	0.20%
Same Sex – Ex Intimate Partner	3	0.12%
Ex Common Law Wife	2	0.08%
Same Sex – Ex Civil Partner	2	0.08%

**Criminal Proceedings Type**		
Charge/further charge	2128	85.12%
First time charged	113	4.52%
Adult caution	95	3.80%
Third and subsequent charge	78	3.12%
Postal charge requisition	41	1.64%
Second time charged	24	0.94%
Summons	16	0.64%
Conditional Caution	2	0.08%
Youth Conditional Caution	1	0.04%
Youth Caution	1	0.04%
Other	1	0.04%
**Case Result***		
Non-Homicide	2263	90.52%
Homicide	237	9.48%

Table S2. Descriptive statistics of continuous features in MPS dataset.

**Feature**	***M***	***SD***	***Median***	***Mode***	***Min***	***Max***
Age of Offender	34.99	11.25	34	30	12	87
Offender Crime Count	0.29	0.78	0	0	0	10
Offender Domestic Violence Count	0.09	0.32	0	0	0	4

Preprocessing

Five essential data modifications were made to convert these features into a format conducive to machine learning: ordinal encoding, one-hot encoding, standardization, the removal of irrelevant features, and the removal of redundant features.[23] First, all ordinal features were converted to numeric values representing their natural order. For example, the risk level of a domestic abuser was encoded as high, medium, small, or unknown, so these categories were converted to the values 3, 2, 1, and 0. Second, nominal categorical features were one-hot encoded into binaries.

Third, the dataset had three continuous features: age, crime count, and domestic violence count. Metric variables are typically standardized via z-scores in machine learning,[23] so age was likewise standardized. Crime counts and domestic violence counts, however, were not standardized. Specifically, most people in the dataset have never committed a crime or an incident of domestic violence; even if they had, it was typically only a couple of episodes at most. Converting these values to z-scores might obfuscate their meaning, given their exceptionally low variance, and thus, they were left unmodified so that they can be treated as ordinal. Fourth, the MPS assigned an arbitrary case ID, so it was dropped due to it being irrelevant. Fifth, there were two redundant features: crime prevalence and domestic violence prevalence. Both crime and domestic violence prevalence measure *whether* someone committed a crime or domestic violence incident; this information is already measured by the crime count and domestic violence count features, so they were accordingly dropped.

Model Evaluation: Key Performance Indicators

Four key performance indicators were used to assess and evaluate the different models: recall, precision, specificity, and Area Under the Curve (AUC). They were selected because they are used to estimate the predictive validity of the various instruments in both the machine learning literature and the domestic abuse screening tool literature.[6,7,24] These key performance indicators were derived from values from a confusion matrix, whose discussion appears below.

Confusion Matrix. A confusion matrix represents a prevalent means of illustrating the performance of a machine learning model,[25] shown in Table S3. It is a two-by-two contingency table in which the model's predicted value is the y-axis. The true value is the x-axis, including true positive count (TP) and true negative count (TN) – the number of homicide predictions the model got right – and false positive count (FP) and false negative count (FN) – the number of homicide predictions the

Table S3. Example confusion matrix. Green shading indicates correct predictions, whereas red shading indicates incorrect predictions.

		Predicted	
		**Homicide**	**Not a homicide**
**Actual**	**Homicide**	True Positive Count (**TP**)	False Negative Count (**FP**)
	**Not a homicide**	False Positive Count (**FP**)	True Negative Count (**TN**)

model got wrong. Percentages can also be displayed alongside counts. These four measurements are the foundation for the key performance indicators described below.

Recall/Sensitivity. Recall is the proportion of homicide cases that the model could flag. For example, if there were 100 homicide cases and the model had a recall score of 0.5, then the model could identify 50 of the 100 cases. Recall is also known as the "true positive rate," and its equation is given by Powers:[24]

(S1) $$Recall/Sensitivity = True\ Positive\ Rate\ (TPR) = \frac{TP}{TP+FN}$$

Precision. Precision refers to the proportion of cases that the model predicted to result in a homicide that actually occurred. For example, if a model had a precision rate of .25, then this means that for every four cases the model flagged as being at risk of homicide, only one will actually be a homicide. The other three cases are false discoveries. For this reason, precision is also known as the mathematical complement of the false discovery rate, and its equation is given by Powers:[24]

(S2) $$Precision = 1 - False\ Discovery\ Rate = \frac{TP}{TP+FP}$$

Specificity/True Negative Rate. Specificity is the proportion of cases that the model predicted to never result in a homicide that was correct. For example, if a model had 100 non-homicide cases and a specificity of .75, then it would interpret 75 of those 100 cases as non-homicides. It is also

known as the true negative rate. It is the mathematical complement of the false positive rate, and its equation appears below:[24]

(S3) $$Specificity/True\ Negative\ Rate = 1 - False\ Positive\ Rate = \frac{TN}{TN+FP}$$

Area Under Curve (AUC). The AUC is a metric traditionally used in assessing medical tests that has become somewhat ubiquitous in machine learning.[26,27] To provide context, each model faces a fundamental trade-off between true positives and false positives. For example, a model may be able to detect more homicides (true positives) if it lowers the decision threshold needed to flag a homicide; a model can be configured to require 10% confidence to flag a homicide rather than 50% confidence. This decision would undoubtedly result in more homicide detections, but it would also result in more false positives—hence the trade-off emerges. This trade-off occurs again if one were to raise the decision threshold – requiring 99% confidence to detect a homicide, for example – albeit the consequences are reversed.

This trade-off can be graphically illustrated through the Receiver Operator Characteristics Curve (ROC), which plots true positives against false positives across various decision thresholds.[28] One can take the area under the curve (AUC) of the ROC graph to summarize the overall quality of this trade-off, where higher values indicate a better trade-off. AUC is occasionally used as a measure of overall performance, and it is calculated as follows, using the true positive rate and false positive rates that are defined in (S1) and (S3):[24,29]

(S4) $$AUC = \sum_{i=1}^{n} \frac{(FPR_i - FPR_{i-1}) * (TPR_i - TPR_{i-1})}{2}$$

Note that FPR and TPR are vectors of false and true positive rates, respectively, across various decision thresholds, with *n* being their length. AbiNader et al. provide guidance on meaningful interpretation of the AUC score: a score of 1 reflects being a perfect classification, 0.5 reflects a

chance classification, 0 reflects a perfect misclassification, and a value of 0.71 or greater reflects an excellent model.[6]

A Note on Model Assessment: Decision Thresholds. The AUC score implies that the key performance indicators – i.e., recall, precision, and specificity – depend strongly on the model's decision threshold, which can be configured. In other words, lowering the model's decision threshold would usually lead to a higher recall at the expense of specificity and vice-versa for raising the threshold. Thus, unless otherwise stated, all machine learning classifiers used a decision threshold that was set to 0.5. In other words, these models would predict a homicide as long as they were at least 50% confident. These confidence scores were probability-like estimates that ranged between 0 and 1, and they were obtained from models via the scikit-learn 'predict_proba' function.[29] This assertion holds for both the initial models and the models produced from the super dataset.

Initial Model Creation

The ten machine learning classifiers that appeared in the main text were used to produce models, and they were created via the following procedure. First, each classifier was trained and evaluated via ten-fold cross-validation with its default hyperparameters, as defined by the scikit-learn Python package.[29] Second, the classifier was optimized for the MPS dataset via a procedure known as hyperparameter tuning. Details of the complete tunning procedure appear later in these supplemental materials and in Table S6.

Third, the hyperparameter tuning produced an optimized classifier, trained on the MPS dataset to produce an optimized model via ten-fold cross-validation. During this procedure, two models were produced: one from the default classifier – the preliminary model - and one from the optimized classifier, the optimized model. Typically, the preliminary model was disregarded while the

optimized model was used to create the super dataset. In other words, the optimized model almost always performed significantly better than the preliminary model, so there was little need to use the worst-performing preliminary model to create the super dataset. However, if the preliminary model performed exceptionally, then it was also used. Each model's recall, precision, specificity, and AUC scores appear below in Table S4.

Table S4. Key performance indicators of all preliminary and optimized models. Asterisks indicate the model was used in the creation of the super dataset. The Gaussian Naïve Bayes had no meaningful hyperparameters to tune, thus only its preliminary model appears.

Model	Recall	Precision	Specificity	AUC
**Ada Boost**				
Preliminary*	0.1505	0.4174	0.9124	0.5315
Optimized*	0.6500	0.1997	0.7203	0.6851
**Decision Tree**				
Preliminary	0.2262	0.1051	0.7638	0.4951
Optimized*	0.6543	0.182	0.6818	0.6681
**Extra Trees**				
Preliminary	0.1719	0.1216	0.8629	0.5174
Optimized*	0.6500	0.2006	0.7212	0.6856
**Gaussian Naïve Bayes**				
Preliminary*	0.9705	0.0975	0.0592	0.5148
**Gradient Boosting**				
Preliminary	0.1505	0.2362	0.8544	0.5025
Optimized*	0.3976	0.1744	0.7256	0.5616
**K Nearest Neighbors**				
Preliminary	0.0754	0.1151	0.9611	0.5182
Optimized*	0.1089	0.1225	0.9368	0.5228
**Linear Discriminat Analysis**				
Preliminary	0.1125	0.0882	0.9084	0.5105
Optimized*	0.5259	0.1621	0.6667	0.5963
**Logistic Regression**				
Preliminary	0.1000	0.0961	0.9389	0.5195
Optimized*	0.5556	0.197	0.6424	0.599
**Random Forest**				
Preliminary	0.1926	0.1367	0.8868	0.5397
Optimized*	0.6154	0.1883	0.718	0.6667
**Support Vector Machine**				
Preliminary	0.0042	0.0125	0.9969	0.5005
Optimized*	0.6908	0.1799	0.6451	0.6679

Super Learner Creation

The Selected models from Table S4 were used to create a super dataset, a dataset of homicide predictions. For the super learner to be complete, however, one final model must intelligently combine these predictions in a way that produces one final homicide prediction. It is difficult to identify the best-performing model *a priori*,[30,31] so multiple models were trained on the super dataset, pitted against each other, and the best-performing model was selected. Because only one of these models can be used in the final super learner, any model created in this section will hereafter be referred to as model candidates.

First, the ten classifiers from the main text were trained on the super dataset to produce a model candidate; these were the preliminary model candidates. Second, these classifiers then underwent the same hyperparameter tuning described in the above subsection, albeit this tuning occurred with the super dataset, not the MPS dataset. The results of this hyperparameter tuning appear in Table S6. Third, these optimized classifiers each produced an optimized model candidate. Recall, precision, specificity, and AUC scores were obtained for all model candidates under ten-fold cross-validation. This yielded 19 model candidates, and of these models, the optimized decision tree performed best. Therefore, it was selected as the final model behind the super learner, the model that intelligently combined all other homicide predictions to produce one final homicide prediction. The performance of each of these model candidates appears in Table S5.

Table S5. Key performance indicators of all preliminary and optimized model candidates, i.e., models that were trained on the super dataset. Asterisks indicate that the model was selected as the super learner's ultimate model.

Model	Recall	Precision	Specificity	AUC
**Ada Boost**				
Preliminary	0.0254	0.0829	0.9881	0.5067
Optimized	0.6918	0.1787	0.6694	0.6806
**Decision Tree**				
Preliminary	0.1475	0.1473	0.9121	0.5298
Optimized*	0.7764	0.1861	0.6443	0.7104
**Extra Trees**				
Preliminary	0.0667	0.2389	0.9841	0.5254
Optimized	0.6875	0.1996	0.7093	0.6984
**Gaussian Naïve Bayes**				
Preliminary	0.5527	0.2106	0.7941	0.6734
**Gradient Boosting**				
Preliminary	0.0759	0.3841	0.9854	0.5307
Optimized	0.4111	0.071	0.3422	0.3766
**K Nearest Neighbors**				
Preliminary	0.0504	0.219	0.9841	0.5172
Optimized	0.1893	0.1986	0.92	0.5547
**Linear Discriminant Analysis**				
Preliminary	0.1043	0.2704	0.9823	0.5433
Optimized	0.6707	0.1995	0.715	0.6928
**Logistic Regression**				
Preliminary	0.0167	0.1167	0.9965	0.5066
Optimized	0.679	0.2016	0.7128	0.6959
**Random Forest**				
Preliminary	0.0460	0.2583	0.9854	0.5157
Optimized	0.7047	0.1904	0.6871	0.6959
**Support Vector Machine**				
Preliminary	0.0000	0.0000	1.0000	0.5000
Optimized	0.6792	0.2068	0.7247	0.7019

Hyperparameter Tuning

The hyperparameter tuning was undertaken via an exhaustive grid search in a computationally powerful computer environment. Specifically, it was launched on a Google Cloud virtual machine with 60 Intel C2-series Cascade Lake CPUs with a 100 GB boot disk and 240 GB memory. The initial models – those trained on the MPS dataset – and the model candidates – those trained on the super dataset – underwent the same hyperparameter tuning procedure, with the only difference being their dataset. All hyperparameter tuning was undertaken via the scikit-learn package in Python, details of which can be found in both its documentation and in Table S6.[29]

Table S6. Classifiers with their default hyperparameters and the results of their hyperparameter tuning. Asterisk indicates that a classifier was trained with optimized hyperparameters taken earlier in the experiment.

**Model with Hyperparameters**	**Description**	**Values Tested**	**Best Value: Initial**	**Best Value: Model Candidate**
**Ada Boost**				
algorithm	Discrete (SAMME) or continuous (SAMME.R) boosting	SAMME, SAMME.R	SAMME.R	SAMME
estimator	Base estimator from which the ensemble is built	None, Random Forest Classifier, Extra Trees Classifier, Optimized Decision Tree Classifier,* Optimized Random Forest Classifier,* Optimized Extra Trees Classifier*	Optimized Extra Trees Classifier	Optimized Decision Tree Classifier
estimators	maximum number of estimators	1, 2, 5, 10, 15, 25, 50, 100	5	1
learning_rate	Weight applied to each classifier	0.01, 0.1, 0.5, 1, 10	0.01	0.01
**Decision Tree**				
Criterion	Formula to measure split	gini, entropy, log loss	entropy	entropy
Splitter	Splitting strategy	best, random	best	best
max_depth	Max depth of tree	None, 3, 5, 10, 20, 30, 40	5	3
min_samples_split	Minimum number of samples to split a leaf	2, 3, 4	2	2
min_samples_leaf	Minimum samples needed to form a leaf	1, 2, 3, 4, 5, 6, 7, 8, 9, 10, 11, 12, 13, 14, 15, 16, 17, 18, 19, 20	19	17
max_features	Caps the number of features when looking for best split by either no cap, the square root of the number of features, or a log base-two of the number of features	None, sqrt, log2	None	sqrt
class_weight	Whether to use weighing formula (C-1)	None, Balanced	balanced	balanced
**Extra Trees**				
n_estimators	Number of trees in forest	5, 10, 100, 500, 1000	500	10
criterion	How to measure quality of split	gini, entropy, log loss	log_loss	entropy
max_depth	Max depth of tree	None, 3, 5, 10, 20, 30, 40	3	3
min_samples_split	Minimum number of samples to split a leaf	2, 3, 4	2	2
min_samples_leaf	Minimum samples needed to form a leaf	1, 2, 3, 4, 5, 6, 7, 8, 9, 10, 11, 12, 13, 14, 15, 16, 17, 18, 19, 20	8	19
features	Caps the number of features when looking for best split by either no cap, the square root of	None, sqrt, log2	None	None

	the number of features, or a log base-two of the number of features			
class_weight	Whether use the weighing formula (C-1) for the entire sample (balanced), the bootstrapped dataset (balanced_subset), or not at all (None)	none, balanced, balanced_subsample	balanced	balanced
**Gradient Boosting**				
Loss	Loss function to be Optimized	log_loss, exponential	exponential	log_loss
Criterion	How to measure quality of split	friedman_mse, squared_error	squared_error	friedman_mse
N_estimators	Number of boosting stages to perform	10, 50, 100, 250, 500, 1000	100	250
Learning_rate	By how much to shrink the contribution of each tree	0.005, 0.01, 0.1, 0.25, 0.5, 1, 10	10	10
max_depth	Max depth of tree	3, 4, 5	4	3
min_samples_split	Minimum number of samples to split a leaf	2, 3, 4	2	2
min_samples_leaf	Minimum samples needed to form a leaf	1, 2, 3, 5	3	1
features	Caps the number of features when looking for best split by either no cap, the square root of the number of features, or a log base-two of the number of features	None, sqrt, log2	log2	sqrt
subsample	Fraction of samples used to fit base learner	0.8, 0.9, 1	0.9	0.8
warm_start	Whether to erase previous solution (false) or use it to fit more estimators (true)	true, false	TRUE	FALSE
**K Nearest Neighbors**				
n_neighbors	Number of neighbors required for each sample	2, 5, 10, 25, 50, 100, 250, 500, 1000	5	2
Weights	Whether to weigh distances between each point uniformly ('uniform') or by the inverse of their distance ('distance')	uniform, distance	distance	distance
P	Power parameter for Minkowski metric	1, 1.5, 2	2	1.5
Leaf_size	Leaf size of BallTree or KDTree	10, 30, 50, 100, 200	10	10
**Linear Discriminant Analysis**				
Solver	Whether to use single value decomposition (svd), least squares solution (lsqr), or eigenvalue decomposition (eigen)	svd, lsqr, eigen	lsqr	lsqr
Shrinkage	Whether to use no shrinkage (none) or Ledoit-Wolf lemma (auto)	auto, None	auto	auto
Tol	Ranking threshold in SVD solver	.00001, .0001, .001, .01, .1, .5, 1	0.00001	0.00001

Priors	Class priors	None, [.5, .5], [.25, .75], [.1, .9]	[.5, .5]	[.5, .5]
**Logistic Regression**				
Solver	Algorithm for optimization	lbfgs, liblinear, newton-cg	lbfgs	lbfgs
Penalty	Whether to add L1, L2 or both (elasticnet) penalty terms	l1, l2, elasticnet	L2	L2
C	Invese of regularization strength: smaller values specify more regularization	.001, .01, .1, .5, 1, 2, 3, 5, 10	0.01	5
Class_weight	Whether to balance class weight via the weighting formula (C-1)	balanced, none	balanced	balanced
Warm_start	Whether to erase previous solution (false) or use it to fit more estimators (true)	true, false	TRUE	
**Random Forest**				
n_estimators	Number of trees in forest	5, 10, 100, 500, 1000	500	10
criterion	How to measure quality of split	gini, entropy, log loss	entropy	entropy
max_depth	Max depth of tree	None, 3, 5, 10, 20, 30, 40	4	3
min_samples_split	Minimum number of samples to split a leaf	2, 3, 4	2	2
min_samples_leaf	Minimum samples needed to form a leaf	1, 2, 3, 4, 5, 6, 7, 8, 9, 10, 11, 12, 13, 14, 15, 16, 17, 18, 19, 20	25	19
max_features	Caps the number of features when looking for best split by either no cap, the square root of the number of features, or a log base-two of the number of features	None, sqrt, log2	None	None
class_weight	Whether use the weighing formula (C-1) for the entire sample (balanced), the bootstrapped dataset (balanced_subset), or not at all (None)	none, balanced, balanced_subsample	balanced_subsample	balanced
**Support Vector Machine**				
C	Inverse of regularization strength: smaller values specify more regularization	.1, 1, 10, 50, 100, 200, 250	10	200
Kernel	Kernel type	linear, poly, sigmoid, rbf	rbf	linear
Class_weight	Whether to balance class weight via the weighting formula (C-1)	balanced, none	balanced	balanced
Gamma	Kernel coefficient for ‘rbf’ , ‘poly’, and ‘sigmoid’. ‘scale’ uses the inverse of the product of the number of features and the X variance, whereas ‘auto’ uses the same equation without the variance	scale, auto	auto	scale

Contextualization

To contextualize the performance of the super learner, it was compared to the MPS current risk assessment procedure, as well as two state-of-the-art domestic homicide forecasting tools: the Danger Assessment and the Lethality Assessment Programme.[7] First, recall, precision, specificity, and AUC scores were extracted from the MPS current risk assessment system. Specifically, within the MPS dataset, there was an ordinal risk-rating system. A risk assessment of 'high' was interpreted to mean that the MPS was flagging a case of being at risk of homicide, whereas the risk assessments of 'medium', 'low', and 'unknown' were interpreted as other decision thresholds. These risk assessments were made according to the Domestic Abuse, Stalking, Harassment and Honour-Based Violence Assessment (DASH) programme guidelines.[21,22] From these values, recall, precision, specificity, and AUC scores were calculated using equations (S1) through (S4). These calculations were unnecessary for the Danger Assessment and Lethality Assessment Programme. Specifically, these tools reported their AUC and specificity scores verbatim, and their recall and precision scores were reported under the names sensitivity and positive prediction rate, respectively. These four indicators were extracted and renamed, enabling a mostly apples-to-apples comparison between the models.

There was one exception to this reporting: The Lethality Assessment Programme did not report its AUC score,[19,20] a crucial measure of model performance, and thus we were left to estimate it. First, Messing and colleagues report the model's performance for one decision threshold, producing a true positive rate (TPR) and false positive rate (FPR) of .93 and .79 respectively, using equations (S1) and (S3). To estimate AUC from these two values, one can first assume that models typically have a TPR and FPR of 0 at the harshest decision threshold and a TPR and FPR of 1 at the most lenient threshold. Second, these numbers can be combined to create a TPR vector of [1 .93, 0] and

a FPR vector of [1 .79, 0], and, using equation (S4), one gets an AUC score of .3745. This AUC score may be flawed: Messing et al. acknowledge that their model was not designed for alternate decision thresholds for near-fatal domestic abuse cases, making AUC a less-than-reliable metric. However, this AUC score is still crucial for the inter-model comparisons, so the estimate will still be used.

Supplemental References

1. London Metropolitan Police Service. Metropolitan Police Service Crime Dashboard. https://public.tableau.com/views/MonthlyCrimeDataNewCats/Coversheet?%3Adisplay_static_image=y&%3AbootstrapWhenNotified=true&%3Aembed=true&%3Alanguage=en-US&:embed=y&:showVizHome=n&:apiID=host0#navType=0&navSrc=Parse.

2. Office for National Statistics. Homicide in England and Wales: year ending March 2019. https://www.ons.gov.uk/peoplepopulationandcommunity/crimeandjustice/articles/homicideinenglandandwales/yearendingmarch2019#how-is-homicide-defined-and-measured (2020).

3. Office for National Statistics. Homicide in England and Wales: year ending March 2020. https://www.ons.gov.uk/peoplepopulationandcommunity/crimeandjustice/articles/homicideinenglandandwales/yearendingmarch2020 (2021).

4. Office for National Statistics. Homicide in England and Wales: year ending March 2021. https://www.ons.gov.uk/peoplepopulationandcommunity/crimeandjustice/articles/homicideinenglandandwales/yearendingmarch2021 (2022).

5. Office for National Statistics. Domestic abuse victim characteristics, England and Wales: year ending March 2022. https://www.ons.gov.uk/peoplepopulationandcommunity/crimeandjustice/articles/domesticabusevictimcharacteristicsenglandandwales/yearendingmarch2022 (2022).

6. AbiNader, M. A., Messing, J. T., Cimino, A., Bolyard, R. & Campbell, J. Predicting Intimate Partner Violence Reassault and Homicide: A Practitioner's Guide to Making Sense of Predictive Validity Statistics. *Social Work* **68**, 81–85 (2023).

7. Graham, L. M., Sahay, K. M., Rizo, C. F., Messing, J. T. & Macy, R. J. The validity and reliability of available intimate partner homicide and reassault risk assessment tools: A systematic review. *Trauma, Violence, & Abuse* **22**, 18–40 (2021).

8. Bland, M. P. & Ariel, B. *Targeting domestic abuse with police data*. (Springer, 2020).

9. Juodis, M., Starzomski, A., Porter, S. & Woodworth, M. A comparison of domestic and non-domestic homicides: Further evidence for distinct dynamics and heterogeneity of domestic homicide perpetrators. *Journal of Family Violence* **29**, 299–313 (2014).

10. Elisha, E., Idisis, Y., Timor, U. & Addad, M. Typology of intimate partner homicide: Personal, interpersonal, and environmental characteristics of men who murdered their female intimate partner. *International journal of offender therapy and comparative criminology* **54**, 494–516 (2010).

11. Dobash, R. E., Dobash, R. P., Cavanagh, K. & Medina-Ariza, J. Lethal and nonlethal violence against an intimate female partner: Comparing male murderers to nonlethal abusers. *Violence against women* **13**, 329–353 (2007).

12. Goussinsky, R. & Yassour-Borochowitz, D. “I killed her, but I never laid a finger on her”—A phenomenological difference between wife-killing and wife-battering. *Aggression and Violent Behavior* **17**, 553-564:553 (2012).

13. Campbell, J. C. Nursing assessment for risk of homicide with battered women. *Advances in Nursing Science* **8**, 36–51 (1986).

14. Campbell, J. C., Webster, D. W. & Glass, N. The danger assessment: Validation of a lethality risk assessment instrument for intimate partner femicide. *Journal of interpersonal violence* **24**, 653–674 (2009).

15. Snider, C., Webster, D., O’Sullivan, C. S. & Campbell, J. Intimate partner violence: Development of a brief risk assessment for the emergency department. *Academic Emergency Medicine* **16**, 1208–1216 (2009).

16. Messing, J. T., Amanor-Boadu, Y., Cavanaugh, C. E., Glass, N. E. & Campbell, J. C. Culturally competent intimate partner violence risk assessment: Adapting the danger assessment for immigrant women. *Social Work Research* **37**, 263–275 (2013).

17. Peterson, K. Learned Resourcefulness, Danger in Intimate Partner Relationships, and Mental Health Symptoms of Depression and PTSD in Abused Women. *Issues in Mental Health Nursing* **34**, 386–394 (2013).

18. Rollins, C. *et al.* Housing Instability Is as Strong a Predictor of Poor Health Outcomes as Level of Danger in an Abusive Relationship: Findings From the SHARE Study. *J Interpers Violence* **27**, 623–643 (2012).

19. Messing, J. T. *et al.* Police departments’ use of the lethality assessment program: a quasi-experimental evaluation. *Washington, DC: National Institute of Justice* (2014).

20. Messing, J. T., Campbell, J., Sullivan Wilson, J., Brown, S. & Patchell, B. The lethality screen: The predictive validity of an intimate partner violence risk assessment for use by first responders. *Journal of interpersonal violence* **32**, 205–226 (2017).

21. Robinson, A. L., Myhill, A., Wire, J., Roberts, J. & Tilley, N. *Risk-led policing of domestic abuse and the DASH risk model.* https://library.college.police.uk/docs/college-of-policing/Risk-led-policing-2-2016.pdf (2016).

22. Richards, L. Domestic Abuse, Stalking and Harassment and Honour Based Violence (DASH 2009-2023) Risk Identification and Assessment and Management Model. (2009).

23. Zheng, A. & Casari, A. *Feature engineering for machine learning: principles and techniques for data scientists*. (O’Reilly Media, Inc., 2018).

24. Powers, D. M. W. Evaluation: from precision, recall and F-measure to ROC, informedness, markedness and correlation. (2020) doi:10.48550/ARXIV.2010.16061.

25. P, A., M, S. K. A., Vignesh, C. C. & S, K. Machine Learning Solution for Police Functions. in *2023 International Conference on Intelligent Data Communication Technologies*

and Internet of Things (IDCIoT) 463–469 (IEEE, 2023). doi:10.1109/IDCIoT56793.2023.10053461.

26. Fawcett, T. ROC graphs: Notes and practical considerations for researchers. *Machine learning* **31**, 1–38 (2004).

27. DeLong, E. R., DeLong, D. M. & Clarke-Pearson, D. L. Comparing the Areas under Two or More Correlated Receiver Operating Characteristic Curves: A Nonparametric Approach. *Biometrics* **44**, 837 (1988).

28. Bewick, V., Cheek, L. & Ball, J. Statistics review 13: Receiver operating characteristic curves. *Critical Care* **8**, 508 (2004).

29. Pedregosa, F. *et al.* Scikit-learn: Machine learning in Python. *the Journal of machine Learning research* **12**, 2825–2830 (2011).

30. Van Der Laan, M. J., Polley, E. C. & Hubbard, A. E. Super Learner. *Statistical Applications in Genetics and Molecular Biology* **6**, (2007).

31. Phillips, R. V., Laan, M. J. van der, Lee, H. & Gruber, S. Practical considerations for specifying a super learner. *International Journal of Epidemiology* **52**, 1276–1285 (2023).
